# High-performance III-V MOSFET with nano-stacked high-k gate dielectric and 3D fin-shaped structure

**DOI:** 10.1186/1556-276X-7-431

**Published:** 2012-08-01

**Authors:** Szu-Hung Chen, Wen-Shiang Liao, Hsin-Chia Yang, Shea-Jue Wang, Yue-Gie Liaw, Hao Wang, Haoshuang Gu, Mu-Chun Wang

**Affiliations:** 1 Department of Electronic Engineering, Minghsin University of Science and Technology, Hsinchu 30401, Taiwan; 2 Faculty of Physics and Electronic Technology, Hubei University, Wuhan, 430062, People's Republic of China; 3 National Nano Device Laboratories, Hsinchu 30078, Taiwan; 4 Department of Materials and Resources Engineering, National Taipei University of Technology, Taipei, 10608, Taiwan; 5 ADATA Technology Company, New Taipei, 23553, Taiwan

**Keywords:** GaAs, High-k, MOSFET, Three-dimensional device, FinFET

## Abstract

A three-dimensional (3D) fin-shaped field-effect transistor structure based on III-V metal-oxide-semiconductor field-effect transistor (MOSFET) fabrication has been demonstrated using a submicron GaAs fin as the high-mobility channel. The fin-shaped channel has a thickness-to-width ratio (*T*_Fin_/*W*_Fin_) equal to 1. The nano-stacked high-k Al_2_O_3_ dielectric was adopted as a gate insulator in forming a metal-oxide-semiconductor structure to suppress gate leakage. The 3D III-V MOSFET exhibits outstanding gate controllability and shows a high *I*_on_/*I*_off_ ratio > 10^5^ and a low subthreshold swing of 80 mV/decade. Compared to a conventional Schottky gate metal–semiconductor field-effect transistor or planar III-V MOSFETs, the III-V MOSFET in this work exhibits a significant performance improvement and is promising for future development of high-performance n-channel devices based on III-V materials.

## Background

Since the transistor speed in circuit consideration is very impressive, III-V compound semiconductors [1] can be treated as potential channel replacement materials for Si in deep nanoprocess integration. III-V materials such as GaAs and InAs possessing higher electron mobility are expected to conduct higher drive current. Conventionally, operation of III-V field-effect transistors (FETs) mainly relies on a Schottky gate structure to modulate channel potential. However, the Schottky gate suffers from high leakage current issue which restrains III-V devices from very-large-scale integration. Metal-oxide-semiconductor (MOS) gate structure used in Si MOSFET is thermodynamically stable and effective for leakage current reduction. In contrary, the lack of a high-quality oxide/semiconductor scheme has limited the applications of III-V devices for decades. Recently, several groups have demonstrated encouraging results in aspects of III-V surface cleaning or pretreatment methods [2,3], growth of insulator on various III-V materials [4,5], as well as realization of III-V MOSFETs [6-11]. Up-to-date III-V MOSFET technologies have demonstrated significant performance enhancement and have achieved low gate leakage [8,10], high channel mobility [7,11], and high drive current [6]. Consequently, it is feasible to produce high-performance MOSFETs using III-V materials. On the other hand, when the scaling of planar Si complementary-symmetry metal-oxide-semiconductor (CMOS) gradually approaches its physical limit, three-dimensional fin-shaped FET (FinFET) device architecture [12-15] is a promising alternate enabling transistor scaling beyond the 22-nm technology node. FinFET [16] structure provides superior control of short channel effects [13]; however, there are only few reports on III-V-based FinFETs [15,17,18]. In this letter, for the first time, a novel III-V MOSFET device technology based on a three-dimensional FinFET structure is reported. Al_2_O_3_ film [19] is used as the gate insulator [4], and submicron GaAs fin is the channel. Both III-V MOSFET and metal–semiconductor FET (MESFET) with a FinFET structure were fabricated, characterized, and evaluated.

## Methods

**Figure 1:**
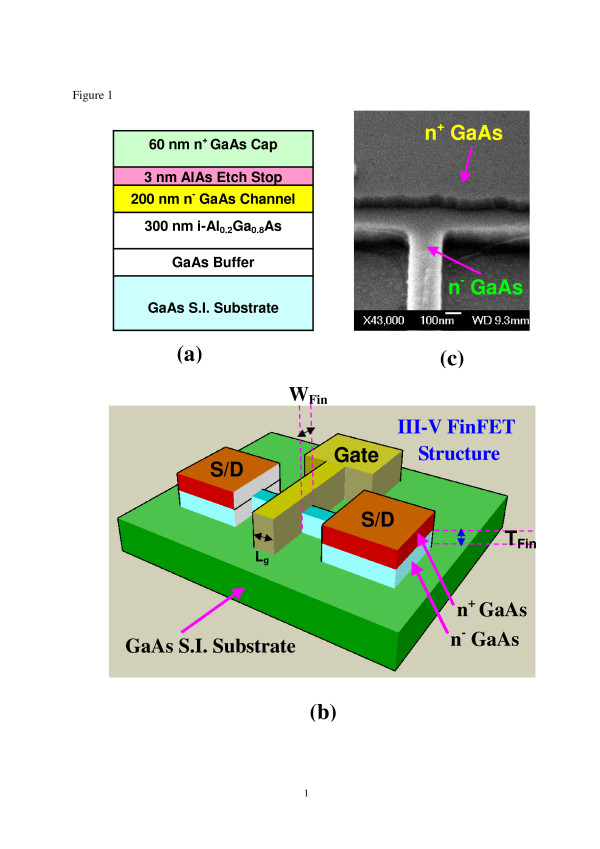
**Schematic diagrams of the III-V MOSFET.** (**a**) Cross section of the epitaxial structure, (**b**) 3D III-V MOSFET with FinFET structure, and (**c**) SEM image of the submicron GaAs fin and the n^+^ GaAs contact region.

GaAs epitaxial wafer grown by molecular beam epitaxy was used as a vehicle for studying III-V-based MOSFETs. The device structure, as shown in Figure 1a, consists of a 300-nm Al_0.2_Ga_0.8_As buffer layer on a semi-insulating (S.I.) GaAs substrate, a 200-nm GaAs channel layer with a doping concentration of 3 × 10^17^ cm^−3^, a 3-nm AlAs etch stop layer, and a 60-nm heavily doped GaAs cap layer at the top. Figure 1b shows a schematic diagram of the III-V MOSFET with a FinFET structure fabricated on the S.I. GaAs substrate. The source/drain regions contain heavily doped GaAs layer for low contact resistance. The gate strip crosses the narrow GaAs fin forming the resultant three-dimensional (3D) FinFET structure. The key fabrication processes for III-V MOSFET and MESFET include removing the GaAs cap layer by wet etching method using citric acid/hydrogen peroxide solution, selectively removing the AlAs etch stop layer to reveal the underneath GaAs channel, and patterning the active GaAs fin region by electron beam lithography. Subsequently, dry etching was performed using inductively coupled plasma to etch down to the buffer layer to form the GaAs fin and simultaneously offer better device isolation. Note that the wet etch process is widely used in mesa isolation step for conventional III-V FETs. However, in this study, a dry etch process was adopted instead to form GaAs fin mainly due to the better integrity of submicron pattern transfer using the dry etch process. As shown in Figure 1c, the edge profile of the submicron GaAs fin is well defined by the dry etch process and good selectivity between n^+^ GaAs/n^−^ GaAs is also achieved by the wet etch process using the AlAs layer as etch stop. AuGeNiAu ohmic contacts were deposited by electron beam evaporation, followed by lift-off process and rapid thermal annealing treatment at 400°C for 30 s. The gate metal is Ti/Au, and the gate length is 0.5 μm. For III-V MOSFET fabrication, there is an additional step of gate insulator growth before the deposition of the gate metal to generate the final MOS structure. The insulator used is an Al_2_O_3_ high-k dielectric. Surface pretreatment prior to dielectric deposition is important to ensure an unpinned interface between the dielectric and GaAs [2-4]. After surface treatment using an ammonia-based solution [3], the wafer was subsequently transferred to an atomic layer deposition system for Al_2_O_3_ deposition. The growth temperature is 300°C, and the thickness of the Al_2_O_3_ is 10 nm. For MESFET, hydrochloric acid solution was used for surface treatment before gate metal deposition. Both fabricated MOSFETs and MESFETs have the same 3D FinFET structure. The thickness (*T*_fin_) and width (*W*_fin_) of the GaAs fins are both 200 nm.

## Results and discussion

**Figure 2:**
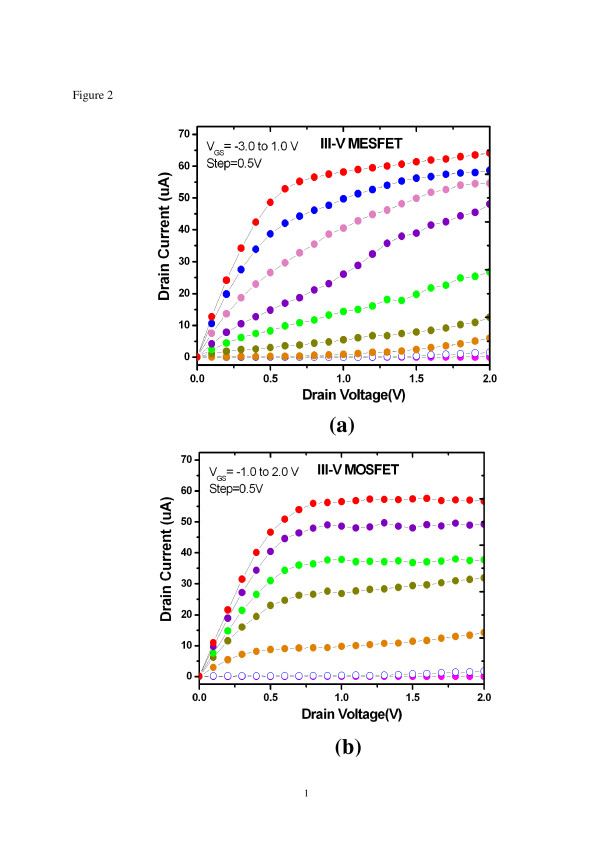
***I ***_**DS **_**-*V ***_**DS**_**characteristics with FinFET structure.** (**a**) 0.5-μm III-V MESFET and (**b**) 0.5-μm III-V MOSFET.

Drain current (*I*_DS_) versus drain voltage (*V*_DS_) curves under different gate voltages (*V*_GS_) of the devices are shown in Figure 2. The threshold voltage (*V*_th_) is −1.5 and −0.25 V for III-V MESFET and MOSFET, respectively. In Figure 2a, a kink behavior was observed. The knee voltage which defines the transition between linear and saturation regions in the normal *I*_DS_*V*_DS_ curve was smeared as the channel is near pinch-off. This phenomenon is related to Fermi level pinning and electron trapping by surface states [20]. A depletion region was created between gate and source/drain electrodes which results in reduced drain output current. When the gate bias is increased, the device behaves more like a typical FET. For the *I*_DS_*V*_DS_ curves of the MOSFET as shown in Figure 2b, the performance was improved. This is mainly due to the deposited Al_2_O_3_ dielectric layer on the surface of the GaAs channel. The Al_2_O_3_ high-k dielectric layer not only acts as a gate insulator, but also plays an important role of surface passivation [21]. The significant performance difference between MOSFET and MESFET implies that devices with a three-dimensional FinFET structure inherently suffer from surface trap issue more seriously than conventional planar devices. This is primarily due to the additional exposed side walls of the fin-shaped channel (i.e., the channel has larger surface-to-volume ratio). Consequently, a good device passivation procedure preventing surface trap-induced effects is indispensable for III-V FETs with a FinFET structure to ensure high device performance.

**Figure 3:**
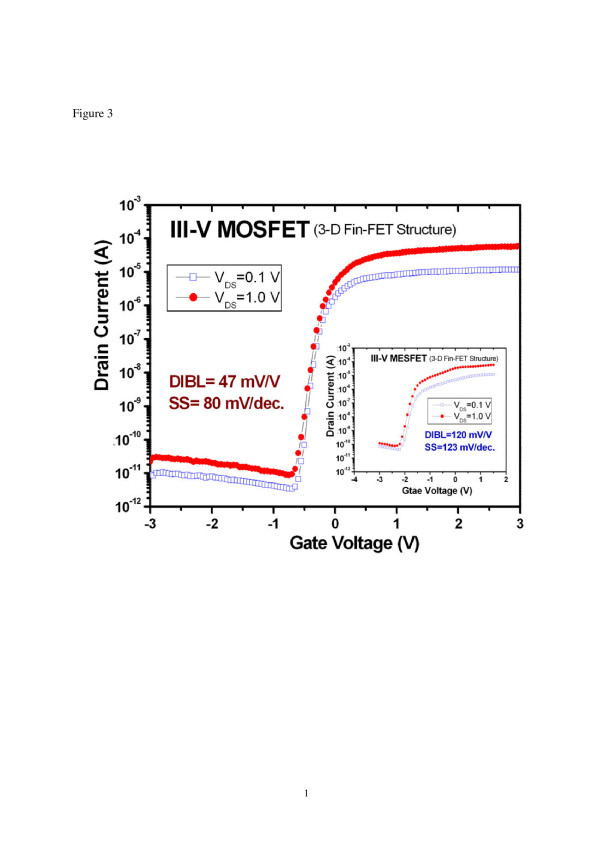
**Subthreshold characteristics of the 0.5-μm III-V MOSFET with FinFET structure.** Inset shows the characteristics of the III-V MESFET for comparison.

The subthreshold characteristics of the devices were also evaluated to further verify the benefit of applying a FinFET structure to III-V MOSFETs. Figure 3 shows the transfer curves of the devices measured at *V*_DS_ = 0.1 and 1 V. Device parameters such as drain-induced barrier lowering (DIBL), on current/off current (*I*_on_/*I*_off_) ratio, and subthreshold swing (SS) were extracted. The calculated DIBL of MESFET is 120 mV/V (shown in the inset of Figure 3), while the value is decreased to 47 mV/V for MOSFET. By introducing a dielectric film, the gate leakage current of the device can be reduced as shown in Figure 3. This is beneficial for improving the *I*_on_/*I*_off_ ratio of the device. The definition of *I*_on_ and *I*_off_ can be found in the literature [22]. The supply voltage *V*_CC_ is 1 V for parameter extraction. The MESFET has an *I*_on_/*I*_off_ ratio of 1.17 × 10^2^, and the ratio is improved significantly to 2.54 × 10^5^ for MOSFET. The SS at *V*_DS_ = 1 V is 123 mV/decade for MESFET and 80 mV/decade for MOSFET. The low SS value of the MOSFET is an indication that the devices have low interface trap density and good gate controllability over the channel [8,23]. These results further demonstrate that MOSFET outperforms MESFET in terms of subthreshold characteristics. As a result, the use of a MOS gate scheme is essential in the performance improvement of the III-V MESFETs. The extracted effective channel mobility in the linear region of the III-V nMOSFET was about 100 cm^2^/V-s using the following expression: $I_{\text{DS}}=\frac{W}{L}\cdot \mu \cdot C_{\text{gate}}\cdot \left(V_{\text{GS}}-V_{\text{th}}-\frac{V_{\text{DS}}}{2}\right)\cdot V_{\text{DS}}$[14], where *μ* is the carrier mobility and *C*_gate_ is the gate capacitance per unit area. The 3D III-V nMOSFET has a total gate width *W*/gate length *L* = 0.6:0.5 μm. The low value of the extracted channel mobility of the 3D III-V nMOSFET was possibly due to the high parasitic access resistance caused by the narrow fin in the source/drain (S/D) regions. Further improvement can be achieved by using a self-aligned S/D process or forming a heavily doped fin region in the S/D extension. In short, the comparison of electrical performance between 3D III-V nMOSFET and 3D III-V nMESFET is presented in Table 1. As shown in Table 1, when compared to conventional planar III-V MOSFETs, the fabricated MOSFET in this work with a FinFET structure exhibits very promising results under low-voltage operation. Although the *T*_Fin_ of the GaAs fin is 200 nm, the SS value of the device with a 0.5-μm gate length is better than the published results of 1 μm × 100 μm planar In_0.2_Ga_0.8_As MOS-high electron mobility transistor [8] which essentially has longer gate length and buried quantum well channel design with higher carrier mobility. The above results further confirm that the III-V MOSFET developed in this work exhibits excellent gate controllability over the channel due to the use of a 3D FinFET structure.

**Table 1:** Electrical performance of 3D III-V nMOSFET and nMESFET with 0.6-μm gate width and 0.5-μm length

	**MOSFET**	**MESFET**
*I*_on_/*I*_off_ ratio	2.54 × 10^5^	1.17 × 10^2^
*I*_on_ (μA) at *V*_GS_ = *V*_DS_ = 1 V	37	58
*V*_th_ (V) at *V*_DS_ = 0.1 V	−0.25	−1.5
SS (mV/decade) at *V*_DS_ = 1 V	80	123
SS (mV/decade) at *V*_DS_ = 0.1 V	68	109
DIBL (mV/V)	47	120

*I*_on_/*I*_off_, on current/off current; *V*_GS_, gate voltage; *V*_DS_, drain voltage; *V*_th_, threshold voltage; SS, subthreshold swing; DIBL, drain-induced barrier lowering; MOSFET, metal-oxide-semiconductor field-effect transistor; MESFET, metal–semiconductor field-effect transistor.

## Conclusions

Measurement and analysis of high-performance III-V nMOSFET are achieved by applying a FinFET structure to device fabrication. The device exhibits excellent subthreshold characteristics and demonstrates significant performance improvement over conventional Schottky gate nMESFET or planar III-V nMOSFETs because of the enlarging channel width, the existing higher channel electron mobility compared with silicon channel and lower channel interface states, as well as the good gate controllability representing the smaller swing value. The three-dimensional III-V nMOSFET device technology developed illustrates great potential and is promising when the CMOS technology is pushed toward more stringent scaling in the foreseeable future.

## Competing interests

The authors declare that they have no competing interests.

## Authors' contributions

The achievement presented here was completed in collaboration among all authors. MCW, WSL, and HW defined the research topic. SHC provided the tested samples. HCY, YGL, HSG, and SJW collected the measurement data or gave this topic some precious advices. All authors contributed to the data interpretation and analysis. MCW and SHC wrote the paper. All authors have contributed to, checked, and approved the final manuscript.

## Authors' information

SHC is an associate researcher at National Nano Device Laboratories, Hsinchu, 30078, Taiwan. WSL is a full professor in the Faculty of Physics and Electronic Technology, Hubei University, Wuhan, 430062, People's Republic of China. HCY is an assistant professor in the Electronic Engineering, Minghsin University of Science and Technology, Hsinchu, 30401, Taiwan. SJW is an assistant professor in the Department of Materials and Resources Engineering, National Taipei University of Technology, Taipei, 10608, Taiwan. YGL is the vice president of ADATA Technology Company, New Taipei, 23553, Taiwan. HW is a distinguished professor from the Faculty of Physics and Electronic Technology, Hubei University, Wuhan, 430062, People's Republic of China. HSG is a full professor in the Faculty of Physics and Electronic Technology, Hubei University, Wuhan, 430062, People's Republic of China. MCW is a full professor in the Electronic Engineering, Minghsin University of Science and Technology, Hsinchu, 30401, Taiwan.
